# Comparative Analysis of Bacterial Diversity between the Liquid Phase and Adherent Fraction within the Donkey Caeco-Colic Ecosystem

**DOI:** 10.3390/ani12091116

**Published:** 2022-04-26

**Authors:** Zhenwei Zhang, Bingjian Huang, Xiaoyuan Shi, Tianqi Wang, Yonghui Wang, Mingxia Zhu, Changfa Wang

**Affiliations:** Liaocheng Research Institute of Donkey High-Efficiency Breeding and Ecological Feeding, Agricultural Science and Engineering School, Liaocheng University, Liaocheng 252059, China; qingyibushuo@163.com (Z.Z.); 17853148163@163.com (B.H.); xiaoyuans2021@163.com (X.S.); qitianwang9797@163.com (T.W.); wyh19920729@163.com (Y.W.); zhumingxia@lcu.edu.cn (M.Z.)

**Keywords:** microbiota, donkey caecum, colon, liquid phase, adherent faction

## Abstract

**Simple Summary:**

The bacteria residing in donkey hindgut are clearly divided into two distinct ecological sites: liquid phase (Lq) and adherent fraction (Ad). Though both the Lq and Ad bacteria play an important role in feed digestion, the Ad bacteria have not previously been specifically sampled or directly compared with the Lq bacteria. The present study was conducted to comparatively analyze the bacterial community composition between the Lq and Ad fraction within the donkey caeco-colic ecosystem. The results showed that the relative abundance of Bacteroidota, Spirochaetota, Fibrobacterota and Patescibacteria in the Ad fraction was greater than the Lq fraction, indicating that bacteria associated with plant biomass are mainly responsible for plant fiber degradation. Regarding the genus level, the liquid phase presented higher abundance in *Lactobacillus* compared to Ad fraction. The activities of enzymes related to fiber degradation were predicted by PICRUSt, and they were also higher in Ad bacteria than Lq. In addition, the bacterial community composition was also distinct within the donkey caecum, ventral colon and dorsal colon. The present study provides evidence that bacteria adherent to feed particles may be better at plant fiber degradation than Lq bacteria due to the greater cellulolytic populations and activities.

**Abstract:**

Donkey hindgut is an enlarged fermentative chamber that harbors a highly complex and extremely abundant community of anaerobic bacteria. It can be divided into two different ecological sites: liquid (Lq) phase and adherent fraction (Ad) colonized by bacteria. However, the Ad bacteria have not previously been specifically collected or directly compared with the Lq bacteria. In the present study, the digesta collected from the caecum, ventral colon and dorsal colon of nine Dezhou donkeys was separated into Lq and Ad fractions. The bacterial community structure was comparatively determined using 16S rRNA gene sequences by Illumina MiSeq. The Ad bacteria had a higher bacterial diversity than Lq bacteria due to the higher Chao and ACE index (*p* < 0.05). The predominant bacteria at the phylum level were Firmicutes (55.4~74.3%) and Bacteroidota (13.7~32.2%) for both the Lq and Ad fraction. The relative abundance of Bacteroidota, Spirochaetota, Fibrobacterota and Patescibacteria in the Ad fraction was greater than Lq (*p* < 0.05), suggesting that bacteria associated with feed particles were mainly responsible for plant fiber degradation. At the genus level, the abundance of *Lactobacillus* in Lq was greater than that in the Ad fraction (*p* < 0.05), indicating that the bacteria in the Lq fraction were better at hydrolyzing readily fermentable carbohydrates. PICRUSt showed that the activities of enzymes related to fiber degradation in the Ad fraction were also greater than Lq. In addition, the hindgut region also had a significant effect on the bacterial community composition. The relative abundance of *Rikenellaceae*_RC9_gut_group, *Clostridium*_sensu_stricto_1, *Christensenellaceae*_R-7_group and norank_*Bacteroidales*_BS11_gut_group was increased (*p* < 0.05) along the donkey hindgut. In summary, the present study provides evidence that bacteria adherent to plant biomass were different to those in the liquid phase within the donkey caeco-colic digesta, and bacteria associated with feed particles may mainly be responsible for plant fiber degradation.

## 1. Introduction

Donkeys are non-ruminant, hindgut fermenting herbivores. They are free-ranging animals of grassland environments, adapted to eat large quantities of high fiber diet to obtain energy and nutrients, which are necessary for bodily processes [1]. Donkey possess an anatomically specialized caeco-colic structure that accommodates a microbial ecosystem consisting of diverse bacteria, archaea and anaerobic fungi that are capable of fermenting and degrading structural polysaccharides of the plant material [2]. It is assumed that as much as 60–68% of the total energy is furnished through volatile fatty acids (VFAs) produced by caeco-colic microorganisms [3].

In recent years, increasing efforts have been devoted to characterizing the equine hindgut microbiota via high throughput sequencing of fecal [4,5,6] and digesta samples [2,7]. Understanding and describing the core microbiota composition in the equine hindgut is essential to provide information for cornerstone taxa and functions present in the caeco-colic ecosystem. Zhao et al. [8] determined bacterial diversity in the caecum of Xinjiang donkeys and reported that *Ruminococcaceae* and *Lachnospiraceae* played a key role in digesting roughage feed. Liu et al. [7] observed that both Firmicutes and Bacteroides are abundant in the hindgut of Dezhou donkeys. A comparative study of gut microbiota in Tibetan wild asses and domestic donkeys on the Qinghai-Tibet plateau was conducted by Liu et al. [6], and they reported that the two dominant phyla Bacteroidetes and Firmicutes in wild asses were significantly higher than that in domestic donkeys. In the distinct anatomical segments of donkey hindgut, there seem to be differences concerning microbial population and fermentative activity. As mentioned above, the microbial community residing in the big caecum and voluminous colon has been analyzed mostly in the liquid phase (Lq) or fecal samples [6,7,8,9]. Little information is available about the bacterial populations in donkey hindgut that are adherent to (the Ad populations) plant biomass.

Considering some similarities, the comparison has mainly been made between the caeco-colic in equine and the rumen in ruminants [10]. It has long been observed that ruminal microbes develop a dynamic biofilm upon digesta particles [11] that possesses more polysaccharidase activity than the planktonic bacteria [12,13]. This indicates that the microbial community separated from the liquid phase and the adherent fraction in the herbivore gastrointestinal tract may be different. Koike et al. [14] sequenced 91 clones of the 16S rRNA encoding genes from ruminal bacteria adherent to feed particles and noted that some of the clones formed distinct and separate clusters relative to the bacteria recovered from the liquid fraction within the two phyla. Until now, knowledge of the biodiversity adherent to plant biomass in the donkey gastrointestinal tract is limited.

Therefore, the present study aimed to comparatively analyze the bacterial community in the donkey caeco-colic ecosystem collected from the liquid phase (Lq) and adherent fraction (Ad). The molecular diversity and the composition of bacteria in Lq and Ad of the different parts of healthy donkey hindgut (caecum, ventral colon and dorsal colon) were elucidated using the high-throughput sequencing of the V3-V4 regions of the 16S rRNA gene.

## 2. Materials and Methods

### 2.1. Animals

Nine Dezhou male donkeys (303 ± 18 kg, 2.5 years old) were enrolled as the donor animals in the present study. The present study was approved by the Institutional Animal Care Committee at Liaocheng University (Permit No. DFG21010103-1). Donkeys were housed in stalls bedded with sandy soil, regularly vaccinated and dewormed. The diet was based on corn straw (3.5 kg of DM/day) along with a commercial concentrate (1.4 kg of DM/day), divided into 2 meals. The composition and nutrient levels of the basal diet for Dezhou donkeys are shown in Table 1. Animals were fed at 07:00 and 19:00 and allowed ad libitum access to water.

Donkeys were slaughtered according to current regulations. They were clinically healthy, with neither clinical disease nor intestinal disturbances having occurred in the past six months. To euthanize donkeys, electronarcosis of 220 V for 20 s was applied. Donkeys were subsequently slaughtered by exsanguination through conventional humane procedures. After exteriorization of the digestive tract, the caecum, ventral colon and dorsal colon were identified and separated for later sample collection.

### 2.2. Collection Procedure of Liquid (Lq) and Adherent (Ad) Phase Samples

The sterile materials were prepared ahead in the laboratory, and the sterile protective suits were equipped to the sampling personnel during the sampling. Digesta samples were collected from each donkey 3 h post feeding after the slaughter. Before the sampling, the caecum, ventral colon and dorsal colon were tied off with sterilized sutures to prevent mixing between neighboring segments. These organs were set separately in the sterile porcelain plates immediately.

Caecum and colon contents were hand squeezed using sterile gloves through four layers of sterile medical cheesecloth to obtain approximately 500 mL of liquid. The liquid fraction (Lq) of each digesta sample was obtained from these filtrates. The microbial cells of Lq samples were harvested by centrifugation at 12,000× *g* for 20 min at room temperature. The adherent bacteria (Ad) were obtained by gently washing 500 g caecum and colon contents (previously retained in cheesecloth) with sterile physiological saline and hand squeezed twice to remove any remaining liquid-associated bacteria. A 100 g subsample of the squeezed solids was then transferred to a sterile centrifuge bottle and 150 mL of sterile phosphate-buffered saline was added to re-suspend the feed particles. The mixture was gently shaken for 30 s, then centrifuged at 350 g for 15 min at room temperature to sediment the plant particles. The supernatant was carefully removed and transferred to a freshly sterile bottle and the Ad bacteria were harvested by centrifugation at 12,000× *g* for 20 min at room temperature. The Lq and Ad microbial cells were resuspended in a minimal volume of Tris-EDTA (pH 8.0) buffer and stored at −80 °C prior to analysis. In total, 54 fractions were prepared (9 animals × 3 segments × 2 fractions/sample).

### 2.3. The DNA Extraction and PCR Amplification

Following the manufacturer’s instructions, genomic DNA of the Lq and Ad microbial cells was extracted using the E.Z.N.A.^®^ soil DNA Kit (Omega Bio-tek, Norcross, GA, USA). The concentration and purity of the resulting DNA extract was checked by a NanoDrop 2000 UV-vis spectrophotometer (Thermo Scientific, Wilmington, DE, USA) with an OD260/280 ratio evaluation and 1% agarose gel, respectively.

The 16S rRNA V3-V4 regions of the ribosomal RNA gene were then amplified by the ABI GeneAmp^®^ 9700 PCR thermocycler (ABI, Arlington, CA, USA) using the following primers: 338F, ACTCCTACGGGAGGCAGCAG; 806R, GGACTACHVGGGTWTCTAAT with 10 ng template DNA. The PCR were carried out in triplicate and using the following thermal cycling conditions: initial denaturation at 95 °C for 3 min, followed by 27 cycles of denaturing at 95 °C for 30 s, annealing at 55 °C for 30 s and extension at 72 °C for 45 s, and single extension at 72 °C for 10 min, ending at 4 °C. The PCR product was determined by electrophoresis on a 2% agarose gel and purified using the AxyPrep DNA Gel Extraction Kit (Axygen Biosciences, Union City, CA, USA) according to manufacturer’s instructions and quantified using Quantus™ Fluorometer (Promega, Fitchburg, WI, USA).

### 2.4. The Illumina MiSeq Sequencing and Sequence Data Processing

Purified amplicons were pooled in equimolar and paired-end sequenced (2 × 300) on an Illumina MiSeq platform (Illumina, San Diego, CA, USA) according to the standard protocols described by Liu et al. [6]. The raw reads were deposited into the NCBI Sequence Read Archive (SRA) database (PRJNA790788).

According to the standard protocols described by Liu et al. [6], the sequence data were demultiplexed, quality-filtered by Trimmomatic and merged by FLASH. All of the sequences were clustered into operational taxonomic units (OTUs), with more than 97% similarity cutoff using the UPARSE version 7.1, and the chimeric sequences were removed. The taxonomy pipeline [15] and the representative sequences were classified into organisms by applying a naive Bayesian model using the Ribosomal Database Project (RDP) classifier version 2.2 [16] based on the SILVA database (version v138). The taxonomic composition for each cluster was evaluated at the phylum and genus level, and the relative abundance of bacterial community was expressed as a percentage.

### 2.5. Statistical Analysis

Data related to bacterial community were analyzed with fraction (Lq and Ad), hindgut region (caecum, ventral colon and dorsal colon) and their interaction (fraction × hindgut region) as the experimental factors having fixed effects using the MIXED procedure of SAS (SAS Inst. Inc., Cary, NC, USA; version 9.4) in two-way ANOVA according to the statistical model in the following equation:

(1) *Y*_ijk_ = *μ* + *F*_i_ + *R*_j_ + (*F* × *H*)_ij_ + *A*_k_ + *e*_ijk_

where *Y*_ijk_ are the dependent variables, *μ* is the overall mean, *F*_i_ is the fixed effect of the fraction (i = 2, Lq and Ad), *R*_j_ is the fixed effect of the hindgut region (j = 3, caecum, ventral colon and dorsal colon), (*F* × *R*) is the fixed effect of the interaction between the fraction and hindgut region, *A*_k_ is the random effect of animals, and *e*_ijk_ is the random residual error. Tukey-Kramer’s test was applied for means comparison. Least square means and standard errors of means were calculated with the LSMEANS procedure of SAS.

The alpha diversity (Chao, Shannon and Simpson index) was calculated in QIIME (version 1.9.1) and graphed by Origin version 8.0 (OriginLab R, Northampton, MA, USA). The OTU rarefaction curves were calculated and plotted in QIIME (version 1.9.1). A principal coordinates analysis (PCoA) with unweighted UniFrac distance at the genus level was plotted, and an analysis of similarity (ANOSIM) of significant characteristics was performed to assess significant differences among hindgut regions. The bacterial community function was predicted using PICRUSt software. Analyses of the bacterial community data and 16S rRNA predicted functional profiles were carried out using the i-Sanger platform (http://www.i-sanger.com/, accessed on 1 February 2022). Differences were declared at *p* ≤ 0.05, whereas 0.05 < *p* ≤ 0.10 were considered to be a trend.

## 3. Results

### 3.1. Alpha Diversity

The Coverage, Shannon, Chao and ACE were included in the present alpha diversity indices (Table 2). Except a tendency for the interaction in the ACE index, there was no interaction between sample fraction and hindgut region in the observed alpha diversity indices. Neither the fraction nor hindgut region had a significant effect on the coverage. The Shannon, Chao and ACE indices were all greater in the dorsal colon than that in caecum and ventral colon (*p* < 0.05). In addition, the Ad fraction had a higher Chao and ACE than Lq by up to 9.4% and 11.7%, respectively (*p* < 0.05).

### 3.2. Bacterial Community Composition

**Figure 1:**
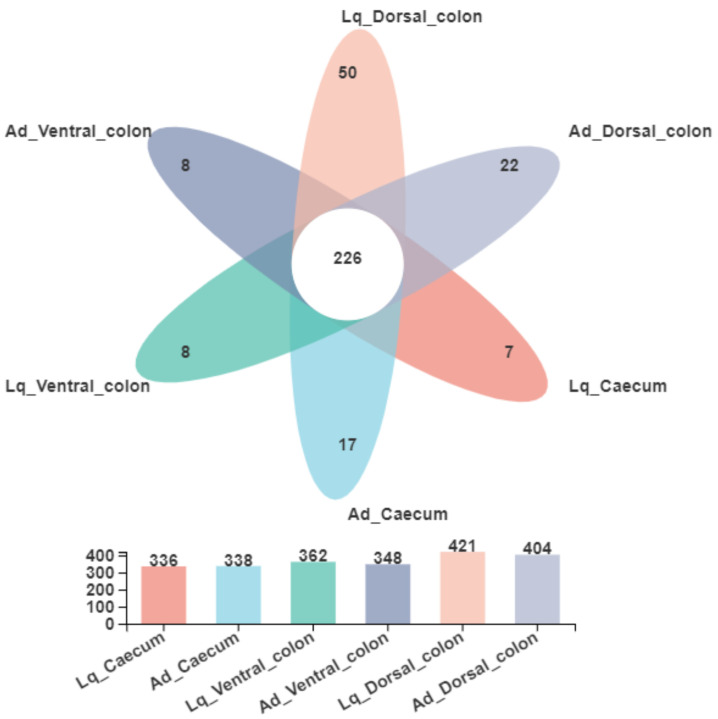
Venn diagram showed the distribution of the bacterial community OTUs at the genus level in the liquid phase (Lq) and adherent fraction (Ad) within the donkey caeco-colic ecosystem.

As shown in Figure 1, a Venn diagram presents the distribution of bacterial community OTUs at the genus level. In the Lq fraction, 336, 362 and 421 OTUs were observed in the donkey caecum, ventral colon and dorsal colon, respectively. In the Ad fraction, 338, 348 and 404 OTUs were observed in each hindgut region. In addition, the shared OTU within all samples was 226.

**Figure 2:**
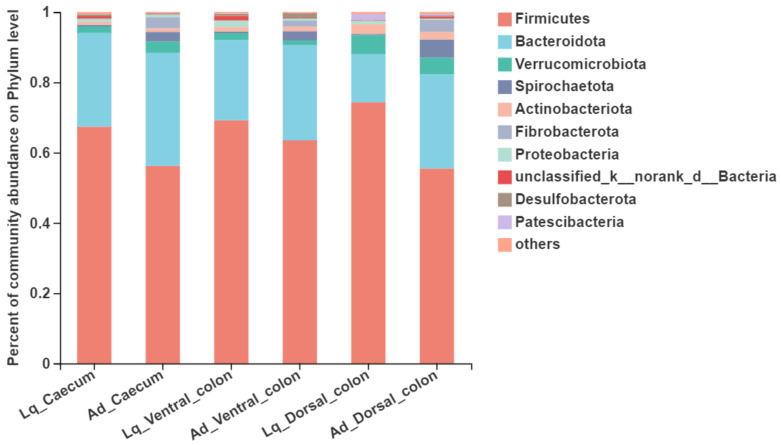
The bacterial community composition at the phylum level in the liquid phase (Lq) and adherent fraction (Ad) within the donkey caeco-colic ecosystem.

At the phylum level (Table 3 and Figure 2), the top five predominant phylum were Firmicutes (55.4~74.3% of the total sequence reads), Bacteroidota (13.7~32.2%), Verrucomicrobiota (1.3~5.5%), Spirochaetota (0.3~5.2%) and Actinobacteriota (0.8~2.8%). No interaction occurred between the sample fraction and hindgut region for the abundance of bacteria at the phylum level. The relative abundance of Bacteroidota, Spirochaetota, Fibrobacterota and Patescibacteria in the Ad fraction was greater than the Lq fraction, but the abundance of Firmicutes in Ad was lower than in Lq (*p* < 0.05). Regarding the influence of the hindgut region, the relative abundance of Verrucomicrobiota, Actinobacteriota and Patescibacteria in the Lq fraction was higher in the dorsal colon than that in caecum and ventral colon (*p* < 0.05). In addition, the relative abundance of Patescibacteria in the Ad fraction was also greater in the dorsal colon compared to the caecum and ventra colon (*p* < 0.05).

**Figure 3:**
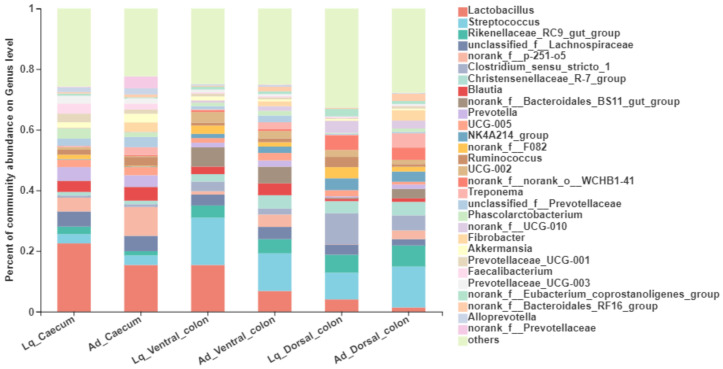
The bacterial community composition at the genus level in the liquid phase (Lq) and adherent fraction (Ad) within the donkey caeco-colic ecosystem.

At the genus level (Table 4 and Figure 3), the top five predominant bacteria were *Lactobacillus* (1.4~22.5% of the total sequence reads), *Streptococcus* (3.1~13.4%), *Rikenellaceae*_RC9_gut_group (1.4~7.0%), unclassified_f_*Lachnospiraceae* (2.1~5.1%) andnorank_f_*p*-251-o5 (0.1~9.6%). There was no interaction between sample fraction and hindgut region for the abundance of bacteria at the genus level. The relative abundance of norank_f_*p*-251-o5 in the Ld fraction tended to be lower than the Ad fraction (*p* = 0.07), but the abundance of *Lactobacillus* and *Clostridium*_sensu_stricto_1 in Lq tended to be greater than in Ad (*p* < 0.10). The hindgut region has a significant effect on the relative abundance of bacterial community at the genus level (*p* < 0.05). The relative abundance of *Lactobacillus*, unclassified_f_*Lachnospiraceae*, norank_f_*p*-251-o5 and *Prevotella* was higher in the caecum than that in the ventral colon and dorsal colon. In contrast, the relative abundance of Streptococcus, *Rikenellaceae*_RC9_gut_group, *Clostridium*_sensu_stricto_1, *Christensenellaceae*_R-7_group and norank_*Bacteroidales*_BS11_gut_group were lower in the caecum than that in colon (*p* < 0.05).

### 3.3. Beta Diversity

**Figure 4:**
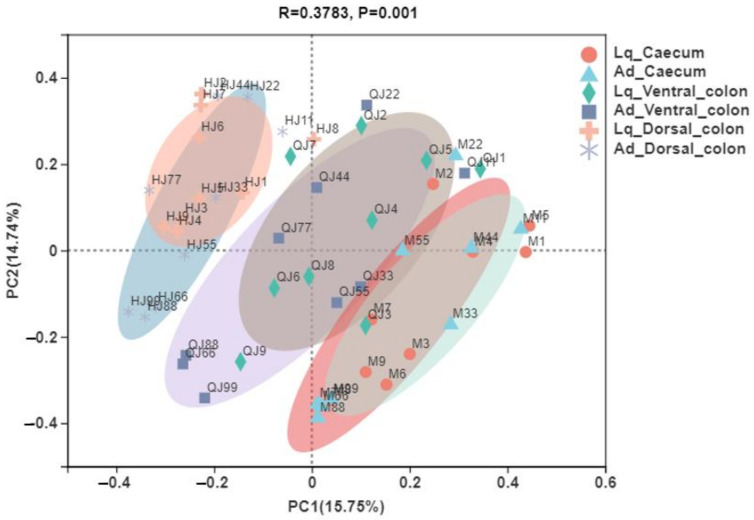
Principal coordinate analysis (PCoA) of bacterial community structures in the liquid phase (Lq) and adherent fraction (Ad) within the donkey caeco-colic ecosystem.

Principal coordinate analysis (PCoA) based on the abund_jaccard metrics was performed to compare the bacterial community structures in the liquid phase (Lq) and adherent fraction (Ad) within the donkey caeco-colic ecosystem (Figure 4). The ANOSIM measurement indicated significant differences on the structure of the bacterial community among donkey caecum, ventral colon and dorsa colon (R = 0.33, *p* = 0.001), while there was no effect on the sample fractions. Principal coordinate 1 and 2 accounted for 15.75% and 14.74% of the total variation.

### 3.4. Variation in Bacterial Function Profiles Analyzed by Reconstruction of Unobserved States (PICRUSt)

**Figure 5:**
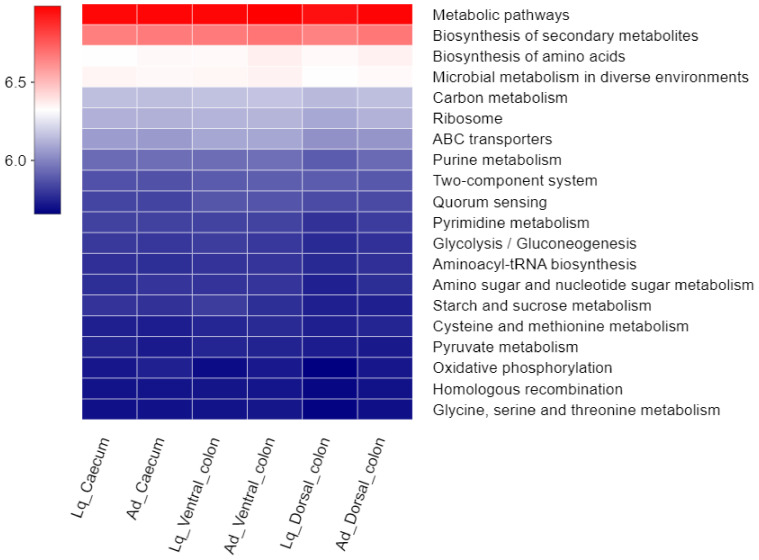
Variation in bacterial function profiles analyzed by PICRUSt. Each row in the heatmap represents a KEGG pathway, each column represents a treatment group. The color gradient in the graph indicates the functional abundance of the KEGG pathway in different treatment groups.

The bacterial functions were enriched in the present samples through PICRUSt (Figure 5). The predicted sequences from the samples revealed the enriched functional features via KEGG pathway analysis. Of these pathways, “Metabolism pathways”, “Biosynthesis of secondary metabolites” and “Biosynthesis of amino acids” were the top three predominant bacterial function profiles in all samples.

### 3.5. Prediction of Microbial Metabolic Function and Enzymatic Activity

The relative abundance of enzymes related to plant fiber degradation was predicted by PICRUSt (Table 5). These enzymes mainly included cellulase (EC: 3.2.1.4), cellulose-1-4-beta-cellobiosidase (EC: 3.2.1.91), endo-1-4-beta-xylanase (EC: 3.2.1.8), xylan-1-4-beta-xylosidase (EC: 3.2.1.37), glucuronoarabinoxylan endo-1-4-beta-xylanase (EC: 3.2.1.136), oligosaccharide reducing-end xylanase (EC: 3.2.1.156), pectinesterase (EC: 3.1.1.11), sialate O-acetylesterase (EC: 3.1.1.53) and feruloyl esterase (EC: 3.1.1.73). There was no interaction between the sample fraction and hindgut region for the abundance of these enzymes. The relative abundance of cellulase (EC: 3.2.1.4) and endo-1-4-beta-xylanase (EC: 3.2.1.8) tended to be lower in Lq than that in the Ad fraction (*p* < 0.05), and the abundance oligosaccharide reducing-end xylanase (EC: 3.2.1.156) and sialate O-acetylesterase (EC: 3.1.1.53) tended to be lower in Lq than in the Ad fraction (*p* < 0.10). However, the abundance of feruloyl esterase (EC: 3.1.1.73) was significantly greater in the Lq phase than that in the Ad fraction. In addition, the abundance of cellulose-1-4-beta-cellobiosidase (EC: 3.2.1.91) and feruloyl esterase (EC: 3.1.1.73) was significantly greater in the dorsal colon than that in caecum and ventral colon (*p* < 0.05). Conversely, the abundance of pectinesterase (EC: 3.1.1.11) was remarkably lower in the dorsal colon than that in caecum and ventral colon (*p* < 0.05).

## 4. Discussion

The donkey hindgut, mainly comprised of the caecum and colon, is an immensely enlarged and anaerobically fermentative chamber [17]. It provides a suitable environment for a large number of anaerobic archaea, bacteria and fungi. Plant structural polysaccharides were fermented and hydrolyzed by these microorganisms to provide 60–68% of total energy by producing VFA [3]. Therefore, it is essential to fully understand the composition and activity of the equine caeco-colic microbiota.

Sadet-Bourgeteau and Julliand [18] noted that the concentrations of the cecal or colonic bacteria were approximately 10^7^ to 10^11^ cells/mL, which represents the majority of the hindgut microbiota in horses. Bacteria are generally most actively involved in dietary fiber degradation. Recently, increasing research has been conducted regarding the bacterial abundance and community composition in equines [3,6,7]. In agreement with the previous study of Liu et al. [6], the present study indicated that the dominant bacteria at the phylum level were also Bacteroidetes and Firmicutes. These microbes account for more than 80% of the total bacteria and facilitate the effective degradation of dietary cellulose and hemicellulose [6,19]. At the genus level, 29 dominant genera with relative abundances exceeding 1% were further displayed. They constituted more than 60% of the total genera reads and were mainly affiliated with the phylum Bacteroidetes (11 genera) and Firmicutes (14 genera).

Fecal samples are commonly applied in the previous studies for bacterial investigation of equine hindgut [20]. However, the present results indicated that the hindgut region appears to influence the abundance of donkey intestinal bacteria. The relative abundance of *Lactobacillus*, unclassified_f_*Lachnospiraceae*, norank_f_*p*-251-o5 and *Prevotella* was greater in the caecum than that in both the ventral colon and dorsal colon. *Lactobacillus* are the common commensals bacteria that have a strong tolerance to acid and produce large amounts of lactic acid in the fermentation of carbohydrates. Generally, the feed ingested by donkeys is first enzymatically digested in the stomach and small intestine through endogenous enzymes, and then the undigested material enters the caecum [21]. Therefore, the caecal microorganisms are first exposed to the rapidly fermentable carbohydrates then to the colon microbes. The soluble carbohydrates and undigested starch in the digesta cannot flow quickly through the caecum and the pelvic flexure, which might promote the abundance of *Lactobacillus* in the caecum. The present results might be different from the previous study of de Fombelle et al. [21], who reported that the concentrations of lactate-utilizing bacteria were lower in the caecum than in other segments of the hindgut. The authors hypothesize that the dietary particles entering the dorsal colon have a lower parietal polysaccharide content due to the longer retention time of coarse particles in the caecum because of the pelvic flexure. However, the bacteria in the caecum may prefer to digest the soluble carbohydrates, undigested starch and protein at first and leave the indigestible particles to the colon in the present study. Future prospective research is needed. *Lachnospiraceae* have the ability to ferment intermediate lactate and acetate to form butyrate, which is essential to the intestinal metabolic balance for the host [22]. *Prevotella* commonly help the breakdown of dietary protein and carbohydrate [23]. The undigested feed from the pre-cecal digestive tract in the donkey also has a large amount of protein that passes into the caecum, enhancing the number of *Prevotella*. In agreement with the study of Liu et al. [6], the relative abundance of *Streptococcus* in the present study was lower in the caecum than that in colon.

In recent years, although some investigations regarding the microbial community profiles in healthy donkey gastrointestinal tract have been implemented [2,7], most samples were collected from the liquid phase, which is equivalent to the present Lq populations. The component of microbiome adherent to feed particles has not previously been specifically sampled or directly compared with the biodiversity present in the Lq populations. Similar to the rumen, the bacterial populations in the donkey hindgut could also be divided into two different ecological sites: liquid phase colonized by bacteria (Lq) and solid phase associated with bacteria (Ad). The differences between Lq and Ad bacteria in community composition and in enzyme activity have indicated that the distribution of bacterial populations is clearly distinct in rumen liquid and solid phases [12]. To our best knowledge, the current study provides evidence that the bacteria adherent to plant biomass are distinctly different to those in the planktonic phase of caeco-colic digesta. In the current study, the relative abundance of Bacteroidota, Spirochaetota, Fibrobacterota and Patescibacteria in the Ad fraction was greater than that in Lq fraction, which is in accordance with the results in ruminants reported earlier by Cheng et al. [11]. Rumen microorganisms associated with the digesta particles possess more polysaccharidase activity than the fluid-borne microbes [12,13]. Therefore, the present higher Ad population in the donkey hindgut could be also related to a preponderant cellulolytic activity. At the genus level, greater populations of *Lactobacillus* and *Clostridium*_sensu_stricto_1 in the Lq phase than in the Ad fraction were observed in the current study. The higher contents of soluble carbohydrates and undigested starch in the caeco-colic liquid fluids may enhance the abundance of lactate-utilizing bacteria (*Lactobacillus*) [21]. Guo et al. [24] reported that *Clostridium*_sensu_stricto_1 is well known as the indispensable regulator of intestinal homeostasis. It can utilize large amounts of dietary nutrients that cannot be digested by the host and produce a great amount of VFAs [24]. In the present study, *Clostridium*_sensu_stricto_1 was also detected as the predominant genus within the donkey hindgut, especially in the dorsal colon. The results of a higher abundance of *Clostridium*_sensu_stricto_1 in Lq than in the Ad fraction suggest that bacterial flora in the liquid phase plays an important role in maintaining the homeostasis of the donkey caeco-colic ecosystem.

The donkey hindgut is indeed colonized by the high concentrations of intensely active bacteria, which have the ability to produce a series of carbohydrate-active enzymes [25]. These enzymes play an irreplaceable role during the plant cell wall breakdown [26]. Based on structural homology and protein sequence, carbohydrate-active enzymes are catalogued into glycoside hydrolases, glycosyltransferases, polysaccharide lyases and carbohydrate esterases [27]. The synergistic actions of all these carbohydrate-active enzymes contribute to the degradation of cellulose, hemicellulose, pectin and lignin present in the feed [28]. Despite its importance, little attention has been focused on the fibrolytic enzyme profile in the donkey hindgut. In the present study, the abundance of fibrolytic enzymes (cellulase, endo-1-4-beta-xylanase, oligosaccharide reducing-end xylanase and sialate O-acetylesterase) related to the degradation of plant fiber were greater in the Ad fraction compared to the Lq fraction. This provides further evidence that bacteria associated with feed particles were better at hydrolyzing cellular wall polysaccharides than the microbes in the liquid phase. The adhesion of bacteria to feed particles is an important factor in successful competition and survival in the donkey hindgut, as well as in the digestion of plant materials. In addition, with the reduction of readily fermentable carbohydrates in the digesta, the relative abundance of pectinesterase in the dorsal colon was lower than that in the caecum and ventral colon. However, the abundance of fibrolytic enzymes including cellulose-1-4-beta-cellobiosidase and feruloyl esterase were increased along the donkey hindgut. Ferulic acid esterases are enzymes that cleave the ester bond between xylans’ primary polysaccharide chain and monomeric or dimeric ferulates, which are essential for complete degradation of plant cell wall polysaccharides [29]. Previous studies have noted that feruloyl esterase is a key lignin-degrading enzyme in hydrolyzing the cross-linkages in the plant cell wall through phenolic compounds [30]. With the digestive transit, the feed particles entering the dorsal colon have a higher lignin content and cross-linking between lignin polymers and polysaccharides, which may improve the feruloyl esterase activity.

## 5. Conclusions

The distribution of bacterial abundance within the donkey hindgut differed between the liquid phase (Lq) and adherent fraction (Ad). The present study provides evidence that bacteria adherent to feed particles in the donkey caeco-colic ecosystem may be better at plant fiber degradation than Lq bacteria due to the greater cellulolytic populations and activities. In addition, the donkey hindgut region also had a significant influence on both the bacterial community composition and the fibrolytic enzyme abundance. It is therefore essential that phase and region of the donkey hindgut are standardized between studies to ensure comparable results.

## Figures and Tables

**Table 1:** Ingredients and nutrient level of the basal diet for Dezhou donkeys.

Items	Basal Diet
Ingredients (g/kg, as-fed basis)	
Corn straw	700
Concentrate	290
Salt	5
Premix ^1^	5
Nutrient level ^2^	
Dry matter, g/kg (as-fed basis)	948.6
Organic matter, g/kg DM	868.8
Crud protein, g/kg DM	168.2
Ether extract, g/kg DM	14.2
Neutral detergent fiber, g/kg DM	462.0
Acid detergent fiber, g/kg DM	257.0
Gross energy, MJ/kg DM	123.8

^1^ The vitamin and trace mineral premix contained (/kg concentrate): Vitamin A 18,000 IU, Vitamin B_1_ 2.5 mg, Vitamin B_2_ 8.0 mg, Vitamin B_3_ 25 mg, Vitamin B_5_ 30 mg, Vitamin B_6_ 0.5 mg, Vitamin B_12_ 50 μg, Vitamin D 3000 mg, Vitamin E 30 mg, Vitamin K 2.5 mg, folic acid 0.5 mg, biotin 90 μg, Cu 50 mg, Fe 200 mg, Mn 50 mg, Zn 200 mg, I 2.0 mg, Se 0.50 mg. ^2^ The nutrient level represents calculated values.

**Table 2:** Bacterial alpha diversity indices in the liquid (Lq) phase and adherent (Ad) fraction within donkey caeco-colic ecosystem.

		Hindgut Region				*p*-Value ^1^		
Items	Fraction	Caecum	Ventral Colon	Dorsal Colon	SEM ^2^	F	R	F × R
Coverage	Lq	0.995	0.992	0.990	0.001	0.273	0.114	0.301
	Ad	0.995	0.991	0.989				
Shannon	Lq	4.16 ^b^	4.02 ^b^	4.72 ^a^	0.211	0.350	0.021	0.540
	Ad	4.15 ^b^	4.48 ^ab^	4.79 ^a^				
Chao	Lq	714.99 ^c^	993.17 ^b^	1196.09 ^a^	57.62	0.050	<0.001	0.192
	Ad	691.17 ^c^	1085.37 ^b^	1426.19 ^a^				
ACE	Lq	699.92 ^c^	988.79 ^b^	1177.42 ^a^	56.30	0.018	<0.001	0.081
	Ad	697.10 ^c^	1089.67 ^b^	1390.98 ^a^				

^1^ F, the effect of fraction; R, the effect of hindgut region; F × R, the interaction between fraction and hindgut region; ^2^ SEM, standard error of means. In the same row, values with different small letter superscripts mean significant difference (*p* < 0.05).

**Table 3:** The relative abundance of bacterial community composition at the phylum level (top 10) in the liquid phase (Lq) and adherent fraction (Ad) within the donkey caeco-colic ecosystem.

		Hindgut Segment				*p*-Value ^1^		
Items	Fraction	Caecum	Ventral Colon	Dorsal Colon	SEM	F	R	F × R
Firmicutes	Lq	74.31	69.21	67.45	6.87	0.038	0.801	0.632
	Ad	56.21	63.45	55.37				
Bacteroidota	Lq	26.57	22.85	13.73	5.35	0.087	0.243	0.680
	Ad	32.21	27.11	26.77				
Verrucomicrobiota	Lq	1.85 ^b^	1.90 ^b^	5.46 ^a^	1.15	0.991	0.010	0.614
	Ad	3.19	1.26	4.80				
Spirochaetota	Lq	0.48	0.44	0.26	0.84	<0.001	0.309	0.200
	Ad	2.82	2.70	5.21				
Actinobacteriota	Lq	0.81 ^b^	1.40 ^b^	2.84 ^a^	0.61	0.745	0.033	0.777
	Ad	1.00	1.38	2.19				
Fibrobacterota	Lq	0.05	0.02	0.01	1.01	0.002	0.637	0.641
	Ad	3.12	1.66	3.47				
Proteobacteria	Lq	0.91	0.90	1.77	0.44	0.091	0.414	0.522
	Ad	0.77	0.63	0.32				
unclassified_k_norank_d_Bacteria	Lq	0.70	1.30	0.15	0.60	0.402	0.776	0.435
	Ad	0.11	0.20	0.58				
Desulfobacterota	Lq	0.52	0.51	0.04	0.32	0.354	0.089	0.232
	Ad	0.18	1.27	0.36				
Patescibacteria	Lq	0.01 ^b^	0.15 ^b^	1.72 ^a^	0.19	<0.001	<0.001	0.103
	Ad	0.03 ^b^	0.03 ^b^	0.36 ^a^				
others	Lq	0.65	0.44	0.57	0.15	0.292	0.443	0.691
	Ad	0.38	0.31	0.57				

^1^ F, the effect of fraction; R, the effect of hindgut region; F × R, the interaction between fraction and hindgut region; SEM, standard error of means. In the same row, values with different small letter superscripts mean significant difference (*p* < 0.05).

**Table 4:** The relative abundance of bacterial community composition at the genus level (top 10) in the liquid phase (Lq) and adherent fraction (Ad) within the donkey caeco-colic ecosystem.

		Hindgut Segment				*p*-Value		
Items	Fraction	Caecum	Ventral Colon	Dorsal Colon	SEM	F	R	F × R
*Lactobacillus*	Lq	22.47 ^a^	15.34 ^ab^	4.03 ^b^	4.65	0.101	0.004	0.805
	Ad	15.38 ^a^	6.77 ^ab^	1.36 ^b^				
*Streptococcus*	Lq	3.07 ^b^	15.64 ^a^	8.75 ^a^	4.84	0.894	0.077	0.720
	Ad	3.21 ^b^	12.42 ^a^	13.42 ^a^				
*Rikenellaceae*_RC9_gut_group	Lq	2.43 ^b^	4.00 ^a^	5.93 ^a^	1.27	0.819	0.004	0.687
	Ad	1.41 ^b^	4.71 ^a^	7.00 ^a^				
unclassified_*Lachnospiraceae*	Lq	4.93	3.66	3.39	1.01	0.777	0.094	0.678
	Ad	5.10 ^a^	4.04 ^ab^	2.13 ^b^				
norank_f_*p*-251-o5	Lq	4.52	1.14	0.11	2.35	0.067	0.050	0.863
	Ad	9.58	4.07	2.88				
*Clostridium_sensu_stricto*_1	Lq	0.71 ^b^	3.18 ^b^	10.25 ^a^	1.39	0.068	0.001	0.138
	Ad	0.85 ^b^	1.97 ^b^	4.93 ^a^				
*Christensenellaceae*_R-7_group	Lq	1.19 ^b^	2.50 ^ab^	4.03 ^a^	0.82	0.265	0.002	0.535
	Ad	1.22 ^b^	4.28 ^a^	4.49 ^a^				
*Blautia*	Lq	3.62	2.47	0.73	1.48	0.445	0.106	0.941
	Ad	4.51	3.94	1.17				
norank_*Bacteroidales*_BS11_gut_group	Lq	0.05 ^b^	6.39 ^a^	0.47 ^b^	1.87	0.697	0.001	0.615
	Ad	0.03	5.52	3.15				
*Prevotella*	Lq	4.57 ^a^	1.55 ^b^	0.41 ^b^	0.83	0.689	0.001	0.588
	Ad	3.90 ^a^	2.04 ^ab^	1.41 ^b^				
others	Lq	52.44 ^ab^	44.17 ^b^	61.90 ^a^	5.41	0.725	0.071	0.660
	Ad	54.83	50.25	58.12				

F, the effect of fraction; R, the effect of hindgut region; F × R, the interaction between fraction and hindgut region; SEM, standard error of means. In the same row, values with different small letter superscripts mean significant difference (*p* < 0.05).

**Table 5:** The abundance of enzymes related to plant cell wall breakdown predicted by phylogenetic investigation of communities by reconstruction of unobserved states (PICRUSt).

		Hindgut Segment				*p*-Value ^2^		
Items ^1^	Fraction	Caecum	Ventral Colon	Dorsal Colon	SEM	F	R	F × R
EC: 3.2.1.4	Lq	11,479	10,556	15,915	2802	0.08	0.32	0.92
	Ad	16,410	15,866	18,995				
EC: 3.2.1.91	Lq	3 ^b^	3 ^b^	8 ^a^	1.6	0.79	<0.01	0.58
	Ad	2 ^b^	1 ^b^	9 ^a^				
EC: 3.2.1.8	Lq	789	565	1104	207	0.07	0.06	0.47
	Ad	1484	774	1274				
EC: 3.2.1.37	Lq	882	781	504	114	0.68	0.27	0.36
	Ad	694	675	665				
EC: 3.2.1.136	Lq	985	396	810	249	0.39	0.35	0.54
	Ad	746	518	326				
EC: 3.2.1.156	Lq	584	377	386	271	0.01	0.69	0.28
	Ad	706	1174	1397				
EC: 3.1.1.11	Lq	972 ^a^	633 ^ab^	340 ^b^	173	0.37	0.02	0.45
	Ad	1079 ^a^	548 ^b^	741 ^b^				
EC: 3.1.1.53	Lq	7207	5373	4368	1441	0.02	0.46	0.83
	Ad	9366	8808	8365				
EC: 3.1.1.73	Lq	1 ^b^	1 ^b^	10 ^a^	1.7	0.05	<0.01	0.10
	Ad	0.5 ^b^	0.3 ^b^	2 ^a^				

^1^ EC: 3.2.1.4, Cellulase; EC: 3.2.1.91, Cellulose-1-4-beta-cellobiosidase (non-reducing end); EC: 3.2.1.8, Endo-1-4-beta-xylanase; EC: 3.2.1.37, Xylan-1-4-beta-xylosidase; EC: 3.2.1.136, Glucuronoarabinoxylan endo-1-4-beta-xylanase; EC: 3.2.1.156, Oligosaccharide reducing-end xylanase; EC: 3.1.1.11, Pectinesterase; EC: 3.1.1.53, Sialate O-acetylesterase; EC: 3.1.1.73, Feruloyl esterase. ^2^ F, the effect of fraction; R, the effect of hindgut region; F×R, the interaction between fraction and hindgut region; SEM, standard error of means. In the same row, values with different small letter superscripts mean significant difference (*p* < 0.05).

## Data Availability

The data are available by sending an email to the corresponding author.
